# Editorial: Recent Advances in Acidophile Microbiology: Fundamentals and Applications

**DOI:** 10.3389/fmicb.2017.00428

**Published:** 2017-03-14

**Authors:** D. Barrie Johnson, Axel Schippers

**Affiliations:** ^1^School of Biological Sciences, College of Natural Sciences, Bangor UniversityBangor, UK; ^2^Geomicrobiology, Resource Geochemistry, Federal Institute for Geosciences and Natural Resources (BGR)Hannover, Germany

**Keywords:** acidophiles, *Acidithiobacillus*, biochemistry, biodiversity, biogeochemistry, extremophiles, genomics

Acidophilic microorganisms thrive in extremely low pH natural and man-made environments such as acidic lakes, some hydrothermal systems, acid sulfate soils, sulfidic regoliths and ores, as well as metal and coal mine-impacted environments. The most widely studied acidophiles, prokaryotes that oxidize reduced iron and/or sulfur, are able to catalyze the oxidative dissolution of metal sulfide minerals such as pyrite (FeS_2_), thereby severely acidify the environment (often to pH <3) in which they thrive. At such low pH values the ferric iron generated by their activities is soluble and serves as the chemical oxidant of sulfide minerals. One the one hand, this is highly beneficial, and is the core process in the biotechnology known generically as “biomining,” where acidophiles are used to facilitate the extraction and recovery of base (e.g., copper, cobalt, nickel, and zinc) and precious metals (principally gold), and also uranium. On the other hand, uncontrolled microbial metal sulfide oxidation in abandoned mines and mine spoils can generate highly noxious waste-waters (acid mine/rock drainage) which, because of their low pH, elevated concentrations of potential toxic metals and metalloids, and high osmotic potentials, pose severe threats to the environment. Recent research, however, has shown that some species of acidophilic microorganisms could also be used not only to mitigate mine water pollution but also to recover metals from acidic waste-waters *via* selective biomineralization.

This *Research Topic* issue comprises 10 original research articles and presents novel data on molecular/ genomic, biochemical, physiological, and applied aspects of acidophilic prokaryotes. These extremophiles may be divided into “extreme acidophiles,” which have pH growth optima at or below pH 3.0, moderate acidophiles, which grow optimally between pH 3.0 and 5.0, and acid-tolerant species which grow optimally above pH 5.0, but which also grow reasonably well at lower pH values. Eight of the papers in this *Research Topic* focus on extreme acidophiles, and most of these describe advances in our knowledge and understanding of the most widely researched class of acidophiles, the *Acidothiobacillia*. New insights into the phylogenetic structure and diversification of *Acidithiobacillus* species revealed by combining analyses of 16S rRNA gene-based ribotyping, oligotyping, and multi-locus sequencing analysis (MLSA) is described in the report of Nuñez et al., who investigated 580 strains of the seven recognized species of the genus (*Acidithiobacillus thiooxidans, A. ferrooxidans, A. albertensis, A. caldus, A. ferrivorans, A. ferridurans*, and *A. ferriphilus*) in their study. Another paper describes how bioinformatic analysis has revealed the existence of five highly conserved gene families in the core genome of the monophyletic genus *Acidithiobacillus* of the class *Acidithiobacillia* (González et al.). Insights into the quorum sensing regulon of *Acidithiobacillus ferrooxidans* revealed by transcriptomic in the presence of an acyl homoserine lactone superagonist analog is described in the report of Mamani et al., while Wang et al. describe how transcriptional analysis has shown that the two-component system RsrS-RsrR regulates the tetrathionate intermediate pathway for thiosulfate oxidation in *Acidithiobacillus caldus*. A novel application of *in situ* spectroscopy carried out by Blake et al. has confirmed that acidophilic iron-oxidizing prokaryotes in different phyla use different electron transfer biomolecules to respire aerobically on soluble iron. Experiments on redox transformations of three transition metals (iron, copper, and chromium) by some *Acidithiobacillus* spp. and two other genera of acidophilic bacteria (*Leptospirillum* and *Acidiphilium*) gave some unexpected results, including the fact that reduction of ferric iron can be mediated under aerobic conditions, and that copper, like iron, can be both oxidized and reduced by acidophiles, though *via* indirect mechanisms (Johnson et al.). Quantifying numbers and activities of acidophiles in natural and anthropogenic environments is becoming increasing important, and Hedrich et al. describe novel quantitative real-time PCR assays for the quantification of *Acidithiobacillus, Leptospirillum*, and *Sulfobacillus* species. When combined with other molecular PCR-based methods, total cell counts and metal sulfide oxidation activity measurements *via* microcalorimetry, this allows highly accurate quantitative monitoring of different microbial species during bioleaching operations.

Another extreme acidophile considered in this *Research Topic* issue is the halotolerant species, *Acidihalobacter prosperus*, which is able to grow and catalyze sulfide mineral dissolution at elevated concentrations of salt (NaCl) and is potentially important for biomining in semi-arid and coastal areas, where only brackish and saline waters are available. The proteomic response of this acidophile to elevated chloride concentrations included the production of osmotic stress regulators that potentially induced production of the compatible solute, ectoine uptake protein, and increased iron oxidation resulting in heightened electron flow to drive proton export by the F_0_F_1_ ATPase. In contrast, *A. ferrooxidans* responded to low levels of chloride with a generalized stress response, decreased iron oxidation, and an increase in central carbon metabolism (Dopson et al.).

The two other papers in this *Research Topic* issue concern contrasting species of moderately acidophilic/acid-tolerant bacteria. Metagenome analysis of strains of the relatively little-researched aerobic and acidophilic iron-oxidizing species *Sideroxydans*, enriched from a pilot plant for the treatment of acid mine drainage, revealed their metabolic versatility and adaptation to low pH (Mühling et al.). Lastly, sequence analysis of the draft genome of the acid-tolerant sulfur-reducer *Desulfurella amilsii*, and comparison to the available genome sequences of other members of the *Desulfurellaceae* family, is presented by Florentino et al.

The manuscripts contained in this *Research Topic* issue illustrate how this particular area of extreme microbiology is continuing to reveal new insights into the molecular biology and evolution of acidophiles, their biochemistries and show their potential for use in new and sustainable biotechnological applications. These fascinating prokaryotes will doubtless continue to reveal new, interesting and potentially highly useful traits as they are further researched.

## Author contributions

All authors listed, have made substantial, direct and intellectual contribution to the work, and approved it for publication.

### Conflict of interest statement

The authors declare that the research was conducted in the absence of any commercial or financial relationships that could be construed as a potential conflict of interest.

